# An Open-Book Approach to Pelvic Dissection for the Male Genitourinary System

**DOI:** 10.7759/cureus.39257

**Published:** 2023-05-20

**Authors:** Jesse Foskey, Mario Loomis

**Affiliations:** 1 Clinical Anatomy, Sam Houston State University College of Osteopathic Medicine, Conroe, USA

**Keywords:** #male genitourinary system, #cadaveric pelvic dissection, #prostate visualization, #pelvic anatomy, #posterior prostate approach, #male urethra

## Abstract

Cadaveric dissections of the male pelvis are predominately conducted using either an anterior approach with the pelvis intact, or via pelvic hemi-section. The anterior approach leaves more tissue in-situ, but has limited visualization of retropubic structures, such as the prostate, seminal vesicles, vas deferens, and urethra. Hemi-section of the pelvis provides increased visualization at the expense of transecting midline structures. This article describes a novel cadaveric dissection, which offers enhanced visualization of pelvic structures in-situ. Using a posterior approach, the pelvis was dissected in an "open-book" manner, which fully exposed the posterior aspects of the prostate, seminal vesicles, ureters, and vas deferens. The delicate neurovascular bundle supplying these structures remained undisturbed. The visualization provided by this dissection correlated well with a coronal MRI of the pelvic region. This open-book dissection provides a novel posterior vantage point of the male genitourinary system, which can help medical students and residents solidify their understanding of anatomical relationships within the pelvis.

## Introduction

The pelvic region is one of the most challenging areas to navigate during cadaveric dissection. Anatomic complexity and variations in cadaver body habitus, such as adipose tissue deposition and pelvic ring diameter, can result in a narrowing of available working space during dissection, as well as a reduction of viewing angles following a completed dissection. These factors create limitations for students and residents while trying to understand the anatomy and anatomical relationships within the posterior pelvis. Standard dissection techniques generally use either an in-situ anterior approach or a hemi-section through the pelvic midline [1-4].

The anterior approach keeps the pelvic anatomy in-situ, which allows for anterior anatomical relationships to be identified [1-5]. However, this approach provides a limited view of the relationships between the retropubic male genitourinary structures. This includes the posterior insertion of the ureters to the bladder, the close approximation of the seminal vesicles and vas deferens as they conjoin to form the ejaculatory duct within the base of the prostate. Other factors influencing the formation of the pelvis such as embryologic development, genetics, environmental exposures, and lifestyle habits, can create variations in the size and shape of the pelvic bowl [6]. These factors can cause reduced visualization of the both the anterior and posterior retropubic structures, likely making these cadavers better candidates for hemi-section dissection.

The traditional midline hemi-section of the pelvis transects the visceral organs and results in two symmetrical halves [1-5]. This approach greatly improves anterior and posterior viewing angles but sacrifices in-situ anatomical relationships that cross the midline. There have been modifications to the midline hemi-section approach, such as the modified midline transection approach described by Steinke et al. [7]. This approach involves dividing the pubic symphysis and sacrum in the median plane after shifting all internal organs to one side, sparing them from transection. Additionally, Hunter et al. performed an en bloc dissection of the pelvic visceral organs followed by a midsagittal hemi-section of the bony pelvis [8]. Both of these modified hemi-section dissections improve visualization without bisecting the soft tissue structures, but they also lose the in-situ relationships of those structures to the bony pelvis. Our posterior open-book dissection of the pelvis improves visualization of pelvic anatomy without soft tissue bisection, but also maintains intact, in-situ pelvic structures for improved correlation with medical imaging and surgery.

## Technical report

**Figure 1:**
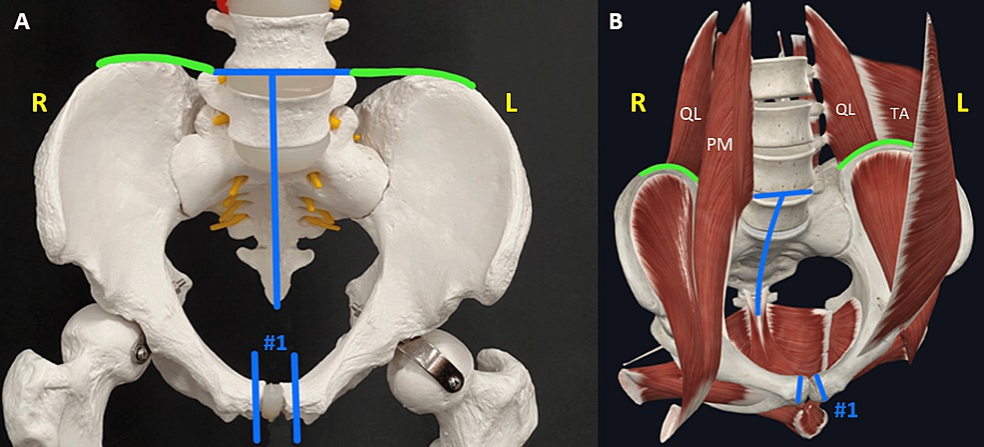
Anterior representation of bone and soft tissue cuts. A) Photograph of anterior pelvis on a skeleton model. Skeleton model -- Courtesy: the Department of Clinical Anatomy at Sam Houston State University College of Osteopathic Medicine. B) 3-D representation of anterior pelvis with relevant musculature. On the right, quadratus lumborum is seen posterior to psoas major. On the left, psoas major was removed from the viewing plane. This is meant to demonstrate that these soft tissue cuts do not extend past the anterior layer of the thoracolumbar fascia. QL, quadratus lumborum; PM, psoas major; TA, transversus abdominis. Image courtesy of Complete Anatomy. #1 represents the bone cuts made approximately one centimeter lateral to the pubic symphysis bilaterally. Blue lines represent bone cuts. Green lines represent soft tissue cuts.

A male cadaver fully embalmed with FAX, a glutaraldehyde arterial fluid containing Entrone for and AD-P, was utilized for the project. With the cadaver supine, a transverse incision was made directly anterior to the pubic symphysis and continued down to the bone. The periosteum was incised, and in the subperiosteal plane, all soft tissue was circumferentially elevated off of the anterior and superior aspects of the pubic symphysis one centimeter from the midline on either side. A reciprocating saw was then used to remove a one-centimeter segment of bone from the pubic symphysis to allow for hinge movement (Figure 1, Cut #1).

**Figure 2:**
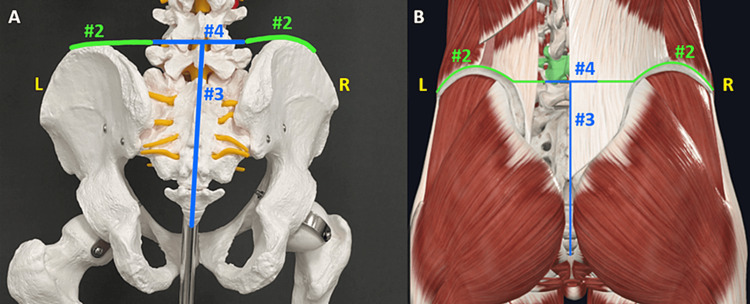
Posterior representation of bone and soft tissue cuts. A) Photograph of skeleton model posterior pelvis. Skeleton model -- Courtesy: the Department of Clinical Anatomy at Sam Houston State University College of Osteopathic Medicine. B) 3-D representation of posterior pelvis with relevant muscles. Posterior thoracolumbar fascia, longissimus thoracis, and multifidus muscles were hidden on the left side of the image to highlight bony landmarks. L4 is marked in green for spinal level orientation. Image courtesy of Complete Anatomy. Blue lines represent bone cuts. Green lines represent soft tissue cuts.

With the cadaver in the prone position, transverse incisions through the soft tissue were made across the midline of the sacrum and extended laterally to the superior aspects the iliac crests, taking care not to damage retroperitoneal structures. These incisions separated the quadratus lumborum, iliocostalis lumborum, external oblique, internal oblique, and transversus abdominis muscles from their insertions onto the iliac crest, and transected the erector spinae muscles as well (Figure 2, Cut #2). Using curved Mayo scissors with the blades slightly open, the soft tissue was separated from the anterior side of the coccyx and sacrum just over the periosteum and a vertical osteotomy was performed through both (Figure 2, Cut #3). The vertical osteotomy was continued through the spinous process and body of L5. Transverse osteotomies were performed through the L4-L5 articular processes with the reciprocating saw, which transected the medial and lateral intertransversarii lumborum muscles and allowed for lateral mobilization and posterior opening of the pelvis (Figure 2, Cut #4). A head block under the hinged pubic symphysis facilitated the lateral pelvic rotation, as did internal rotation of the lower extremities.

**Figure 3:**
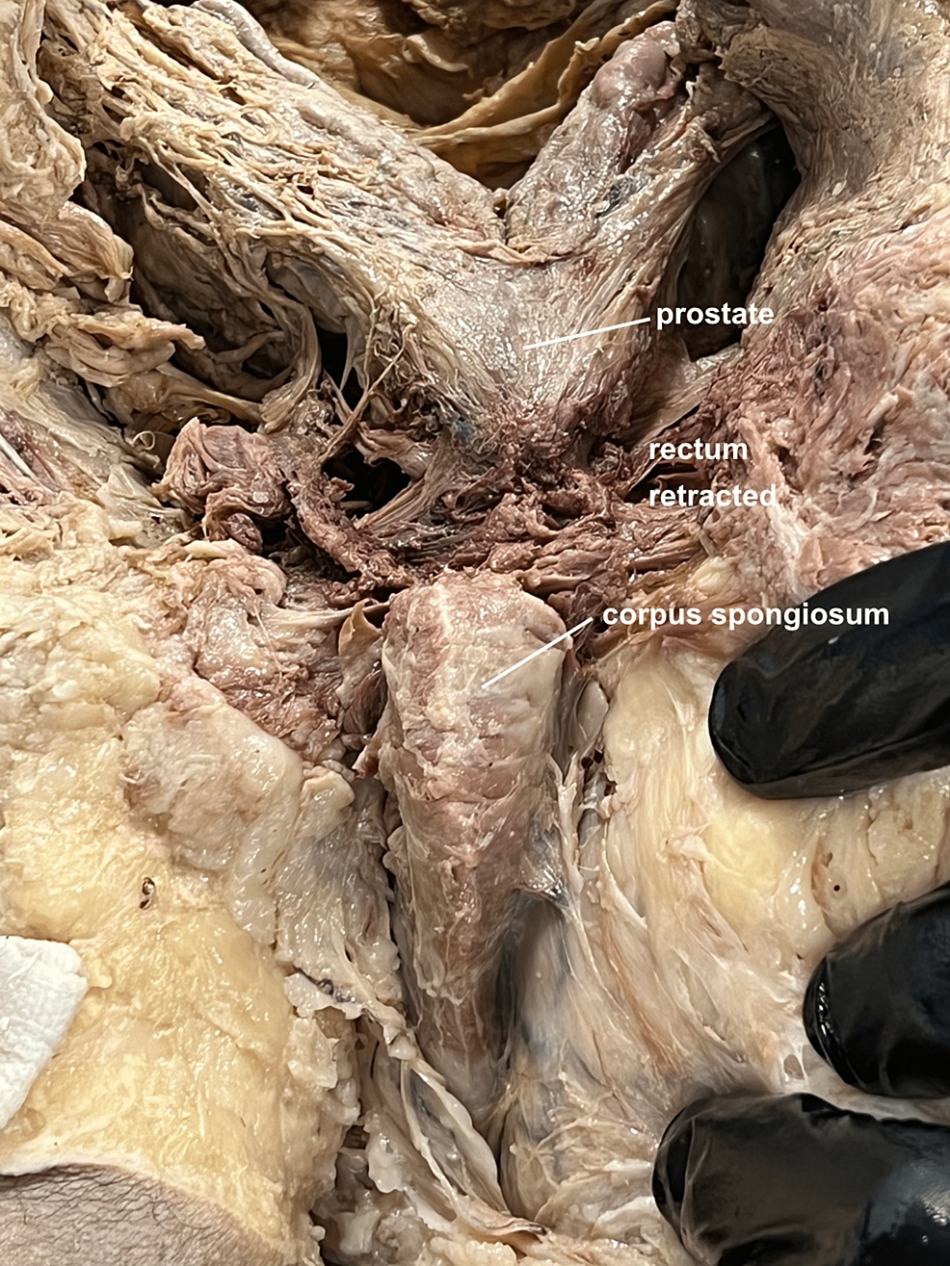
Posterior view of prostate. Bisecting the sacrum and opening the pelvis hinged on a divided pubic symphysis, a posterior view of the prostate is seen after retracting the rectum to the right.

Neurovascular structures, such as the superior and middle rectal arteries and veins, and the superior rectal, middle rectal, and inferior hypogastric nerve plexuses were mobilized sufficiently to retract the rectum laterally, allowing for visualization of the retropubic structures (Figure 3).

**Figure 4:**
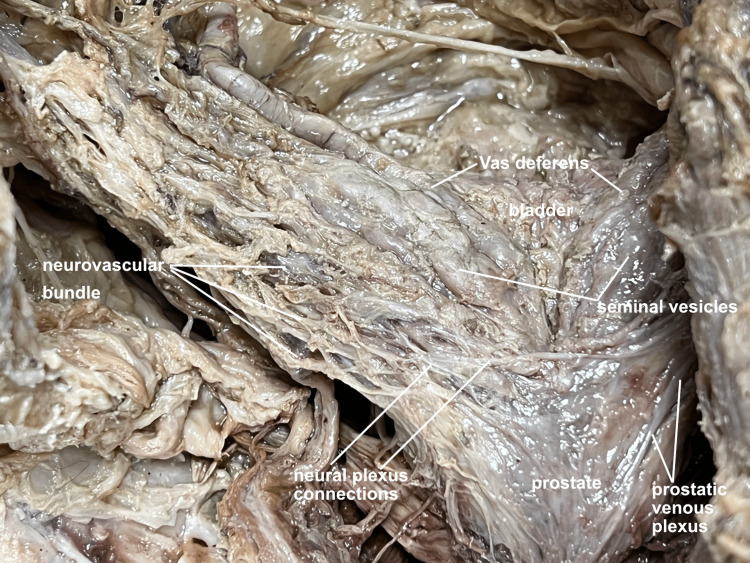
Neurovascular bundle. A closer image taken of the neurovascular bundle posterior to the prostate, seminal vesicles, vas deferens, and bladder. The middle rectal nerve plexus and inferior rectal nerve plexus give rise to the vesical nerve plexus and prostatic nerve plexus, respectively. These plexuses connect just posterior to the seminal vesicle and ampulla of the ductus deferens. The inferior vesical and inferior rectal veins give rise to the vesical and anorectal venous plexus, respectively, which combine to form the prostatic plexus just lateral to the prostate. The inferior vesical artery (not shown) lies on the inferolateral aspect of the bladder. Its branches run deep to the vesical and prostatic venous plexuses as they cross over the posterior aspect of the seminal vesicle to reach the lateral prostate. This artery supplies the posterior aspects of the prostate, seminal vesicles, and the posteroinferior aspects of the bladder. The vas deferens is supplied by the artery to ductus deferens (not shown), a branch of the superior vesical artery (not shown). These arteries which are located on the superolateral aspect of the bladder, which are better appreciated from an anterior view.

The inferior floor of parietal peritoneum was appreciated and could be maintained anteriorly. The neurovascular bundle posterior to the prostate was likewise appreciated and preserved (Figure 4).

**Figure 5:**
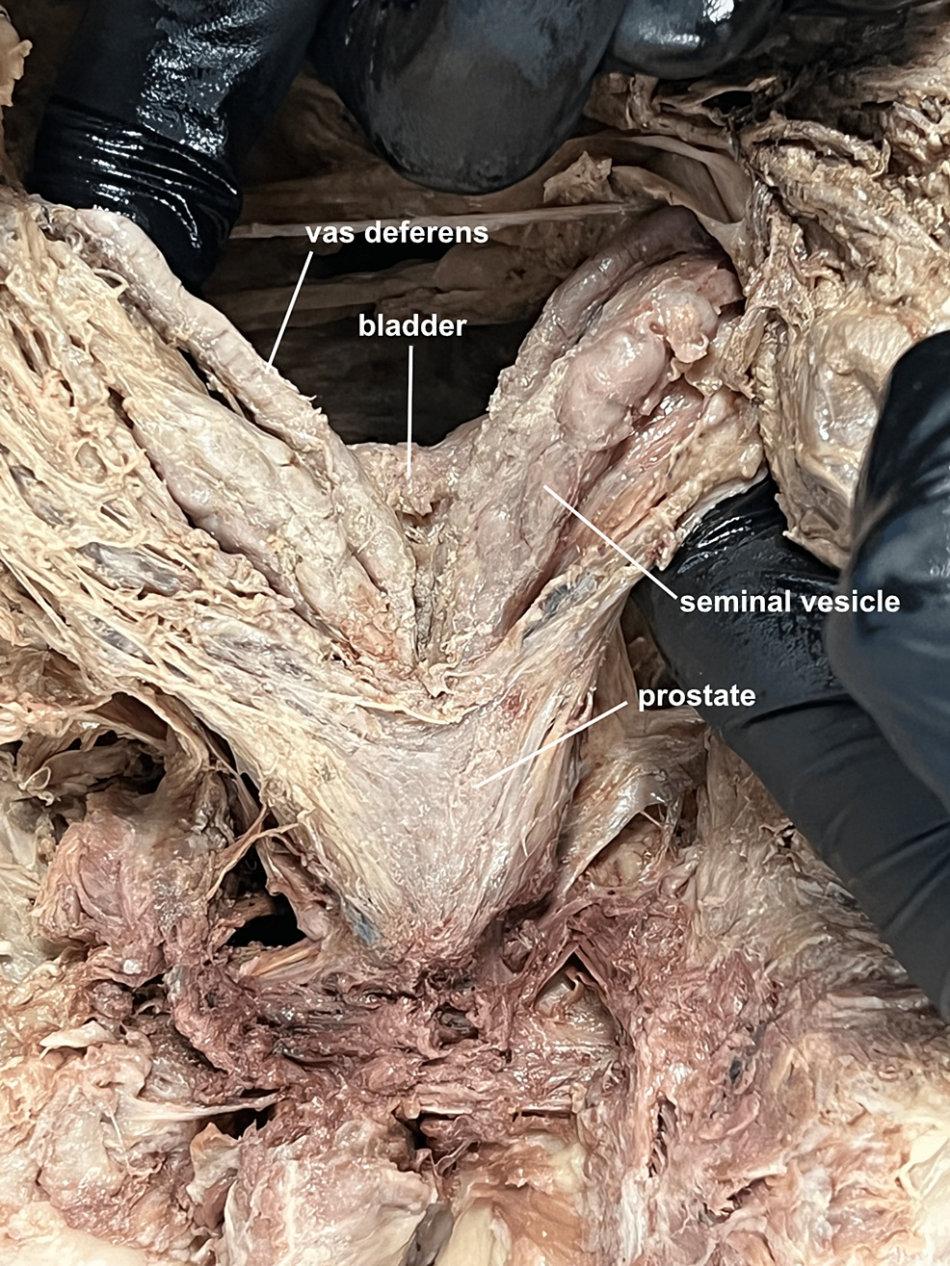
Posterior view of prostate, vas deferens, and seminal vesicle. The bladder, vas deferens, seminal vesicles, and prostate are viewed in-situ from a posterior perspective.

The in-situ relationships of the bladder, prostate, seminal vesicles, and vas deferens were clearly visualized (Figure 5).

**Figure 6:**
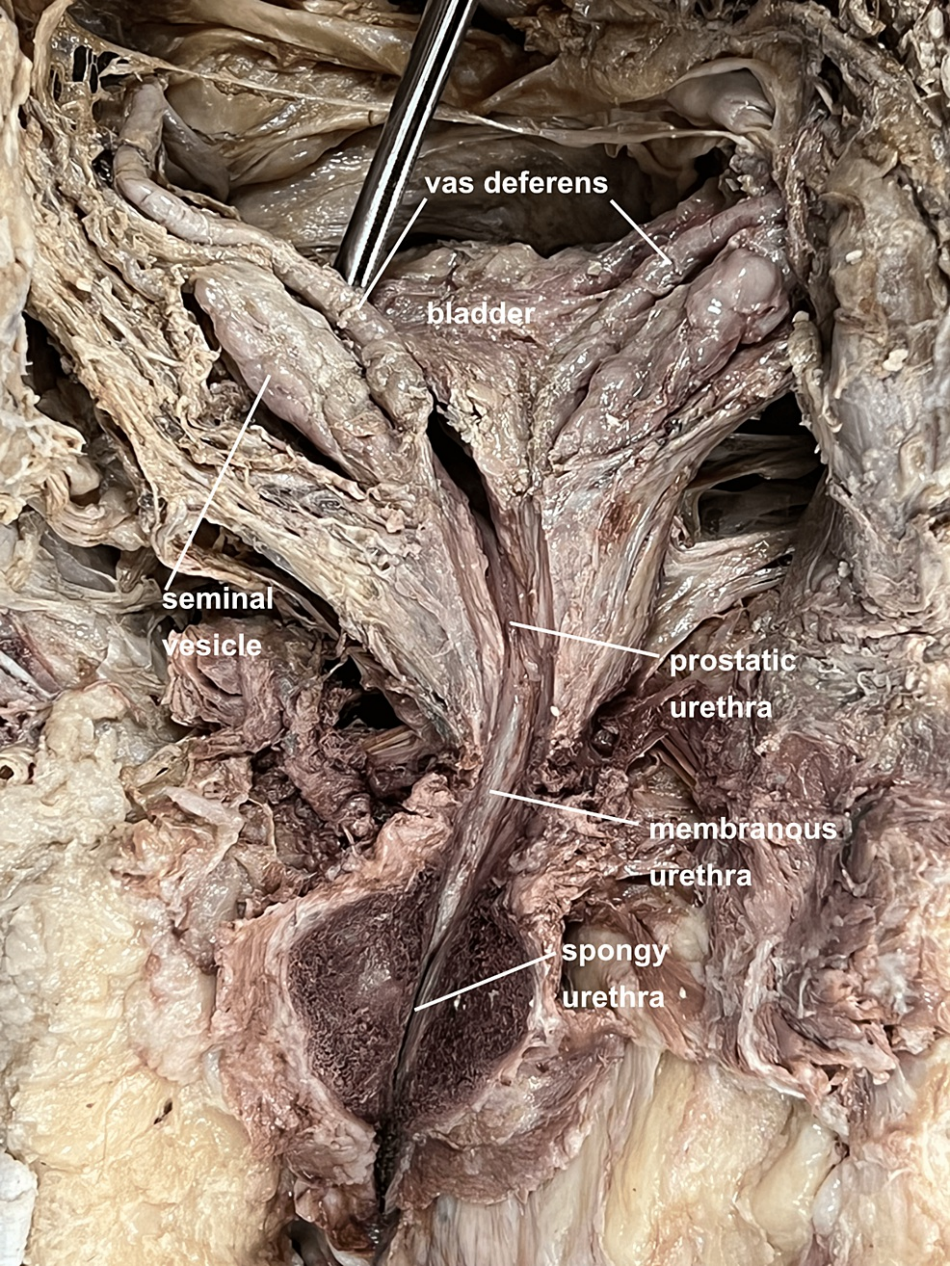
Posterior view of male urethra. By passing a foley catheter, it is possible to precisely open the urethra along its length.

A Foley catheter was passed up to the prostate. At this point, the anus and rectum were further mobilized laterally, and the midline perineum incised so that the posterior aspect of the urethra could be palpated and followed from prostate down to corpora spongiosum. A midline incision was made into the urethra which was then followed proximally and distally under direct vision (Figure 6).

**Figure 7:**
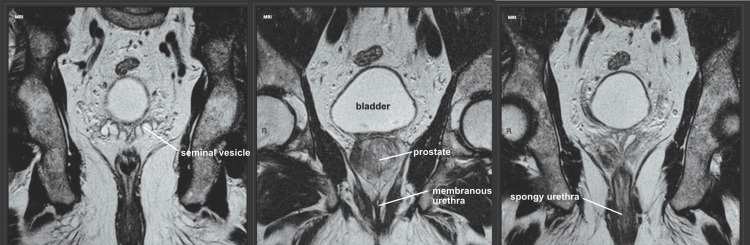
Coronal pelvic MRI correlation. Seeing the pelvic anatomy from this perspective can help medical students and residents orient themselves when viewing diagnostic imaging such as this coronal T2 MRI of the pelvis (coronal T2 MRIs used with permission, courtesy of Ian Bickle, <a href="https://radiopaedia.org/">Radiopaedia.org</a>. From the case <a href="https://radiopaedia.org/cases/82325">rID: 82325</a>) [9].

The posterior open-book pelvic dissection provided several benefits including an improved appreciation of the in-situ relationships of the bladder, prostate, seminal vesicles, and vas deferens (Figures 3-5). Though the sacrum is disarticulated in the midline, the approach is delicate enough to not disturb the anterior peritoneal floor, allowing for a unique visualization of the fragile neurovascular bundles of the prostate (Figure 4). Additionally, passing a catheter from the spongy to the prostatic urethra facilitates the ability to create a precise opening of the urethra along its entire length (Figure 6). This in-situ posterior view correlated very well with coronal MRI images of the region (Figure 7).

## Discussion

Cadaver dissections are expensive and time consuming by nature, which can present logistic, and scheduling challenges, particularly in the context of modern medical training [10]. The standard anterior approach and sagittal hemi-section approach to pelvic dissection are time-tested and efficient means to teach pelvic anatomy. The posterior open-book dissection is not intended to replace that standard. However, when incorporated as a supplemental prosection, the posterior open-book approach can provide the additional insight and perspective that many students need to fully appreciate the interrelationships of pelvic structures. Steinke et al. described this beautifully by stating “Such understanding is achieved by careful dissection of the body, its compartments, the muscles, and the associated neurovascular bundles” [7]. This principle was the inspiration for this research as it is particularly relevant for surgical specialties.

Currently, standard pelvic dissections use either an anterior approach or a sagittal hemi-section approach. The anterior approach begins at the superior aspect of the pelvis, around the level of the thoracic aorta bifurcation, and ends at the perineal floor [1-4]. This maintains the anterior pelvic anatomy in-situ, but it is difficult to visualize. The sagittal hemi-section approach bisects the sacrum and lower lumbar spine, coupled with a lateral transverse cut to allow separation of the leg from the body [1-4]. This improves visualization but sacrifices in-situ relationships at the midline. A thorough posterior view of the pelvis has traditionally required this sacrifice. Steinke et al. and Hunter et al. have described modifications to the hemi-section, in which they keep midline structures intact [7-8]. These approaches involve either dissecting the pelvic soft tissues off to one side or removing the soft tissue en bloc, followed by mid-sagittal hemi-section of the pelvis. These modifications improve visualization and keep the midline structures intact, but sacrifice the normal appearance afforded by lateral structural support. Our posterior open-book dissection provides a thorough posterior view with intact midline structures while maintaining the lateral support necessary for normal bilateral appearance.

A noticeable shortcoming that could be experienced during this dissection is the cadaver’s rectum being full of fecal material. This will cause the rectum to balloon in size and reduce its ability to be laterally displaced. This can limit the exposure of the prostate, seminal vesicles, ureters, and vas deferens, and increase the difficulty of the dissection. One of the limitations regarding this research is the sample size as this dissection was performed on a single male cadaver. However, this does provide opportunity for continuation of this research. This dissection could be expanded for use on female cadavers, which may offer novel posterior vantage points of the vesicouterine and rectouterine pouches. Direct continuation of this research could involve anatomists and medical students recreating this dissection, which would help generate data and build a consensus to determine its effectiveness as an anatomy learning tool.

A multidomain, global assessment survey of surgical subspeciality program directors found that 26% of new surgical fellows could not recognize anatomical planes [11]. The anatomical planes in the pelvis can be particularly difficult for students and residents to learn. From a surgical perspective, pelvic dissection using an anterior approach highlights relevant anatomical landmarks for open surgical procedures, such as a hernia repair using the Stoppa approach or surgical management of an acetabular fracture using the pararectus approach [12-16]. The different perspective from our open-book dissection approach, coupled with the anterior and hemi-section approaches, can supplement students’ three-dimensional understanding of the region. Correlating MRIs with the in-situ cadaver anatomy can also improve appreciation of such imaging. Visualizing the male urethra as it runs from the bladder through the corpora spongiosum can also help solidify the three-dimensional relationships around the male urethra. If future studies demonstrate a positive impact on medical student learning, this research may be further expanded to be used by urologic and general surgery residents. Completion of this dissection could help these residents prepare to assist surgeons operating in or around Denonvilliers’ fascia or the mesocolon [17-19]. 

## Conclusions

In summary, the open-book pelvic dissection is a novel approach which has not been described in current literature. This approach offers a unique view of in-situ pelvic structures and relationships, allowing for improved appreciation of pelvic tissue planes and neurovascular networks. Additionally, the male urethra can be precisely dissected along its entire length with relative ease. This dissection demonstrated clear anatomical correlation with coronal MRIs of the pelvic region. When supplemented alongside standard anterior and hemi-section dissections of the male pelvis, we postulate that this dissection can help medical students and residents solidify their three-dimensional understanding of this relatively complex anatomy.
